# Antimicrobial Agents-induced Hypokalemia: A Possible Causality Association

**DOI:** 10.5005/jp-journals-10071-23148

**Published:** 2019-04

**Authors:** Harmeet Singh Rehan, Priyanka Hotha

**Affiliations:** 1,2 Department of Pharmacology, Lady Hardinge Medical College, New Delhi, India

**Keywords:** Adverse drug reaction, Antimicrobial agents, Hypokalemia

## Abstract

**Background:**

Drugs including some of the antimicrobial agents (AMAs) can cause mild to severe intensity of hypokalemia, which leads to cardiac, muscular, renal, gastrointestinal, and metabolic manifestations.

**Objective:**

To explore the possible association of AMAs use and the development of hypokalemia as an adverse drug reaction (ADR).

**Methodology:**

Retrospective analysis of spontaneously individual case safety reports (ICSRs) received during January 2015 to September 2017 for any reduction in serum potassium levels following the use of AMAs. Such ICSRs were further analyzed for age, gender, seriousness and severity of hypokalemia, outcome, concomitant drugs, management of hypokalemia, and causality assessment using WHO-UMC causality assessment scale.

**Result:**

Out of total 2,880 spontaneous ICSR, 53 had report title of hypokalemia. In almost half of these (27) ICSRs, AMAs were suspected to induced hypokalemia. Ceftriaxone (24.5%) and azithromycin (10.5%) were most suspected AMAs. Females (74.19%) aged between 21 years and 40 years experienced more AMA induced hypokalemia. The mild, moderate, and severe hypokalemia was present in 53.8%, 40.7%, and 7.4% of ICSRs, respectively. Drug–drug interaction of AMA with either furosemide, hydrocortisone and/or deriphyllin was present in six ICSRs. Causal association of all the ICSRs with AMA induced hypokalemia was possible.

**Conclusion:**

Antimicrobial agents (especially ceftriaxone and azithromycin)-induced hypokalemia alert needs to be investigated. Further, healthcare professionals are advocated to take caution by monitoring serum potassium levels routinely for such patients.

**How to cite this article:**

Rehan HS, Hotha P, Antimicrobial Agentsinduced Hypokalemia: A Possible Causality Association. Indian J Crit Care Med 2019;23(4):175-177.

## INTRODUCTION

Intra- and extracellular homeostasis of potassium is important to maintain physiological function of several human cells including cardiac muscle, skeletal muscle, and nerve cells.^1^ Increase and decrease of serum potassium levels may be potentially fatal.^2^ Hypokalemia may result from the shift of potassium ions from the extracellular to the intracellular space, increased potassium excretion in urine, and/or poor potassium intake. Among the hospitalized patients, almost 20% of the patients may develop all cause hypokalemia leading to cardiac, muscular, renal, gastrointestinal, and metabolic manifestations.^3^ In addition, few drugs may also precipitate hypokalemia. Hypokalemia associated with antibiotic use is rare but it is a recognized complication of dicloxacillin, ampicillin,^4^ amphotericin B,^5^ aminoglycosides,^6^ and penicillin^7^ especially when administered to patients with renal or hepatic insufficiency. Recently, Pharmacovigilance Programme of India (PvPI) has also issued a drug alert that piperacillin-tazobactam may induce hypokalemia.^8^ In view of this, AMAs use dependent hypokalemia may concern the clinicians. Hence, this retrospective analyze of spontaneously ICSR was carried out to explore any possible association between AMA use and the development of hypokalemia.

## METHOD

All the spontaneous ICSRs with suspected drugs-induced hypokalemia including AMAs received at ADR monitoring center (AMC) under the aegis of PvPI between January 2015 to September-2017 were analyzed. ICSRs without the mention of serum potassium values were excluded from the analysis. The severity of hypokalemia was graded as mild (3–3.5 mEq/L), moderate (2.5–3 mEq/L), and severe (below 2.5 mEq/L).^9^ ICSRs with AMA-induced hypokalemia were further analyzed for patient information (age and gender), suspected adverse reaction (seriousness, severity, outcome, and management), suspected medication (concomitant drugs and drug–drug interactions), and causality assessment. Causality assessment between suspected AMA and hypokalemia was assessed by using WHO-UMC causality scale.^10^ Data were presented in numbers and proportions.

## RESULT

A total number of 2,880 spontaneous ICSRs were received by the AMC during the study period. Of these, 53 ICSRs contained 120 drugs, which were suspected to induce hypokalemia, with an average of 2.26 suspected drugs per ICSR. Further analysis revealed that there were 27 ICSRs which contained 57 AMA and were suspected to cause hypokalemia. The possible AMA-induced hypokalemia was 0.9% of study. AMAs (47.5%) were the most frequently alleged to cause hypokalemia followed by antineoplastic agents (11.6%) and corticosteroids (9.1%) (Table 1).

**Table 1:** Suspected groups of drugs alleged to cause hypokalemia in 53 ICSRs with a total of 120 drugs

*S. No*	*Group of drugs*	*Number (%)*
1	Antimicrobial Agents	57 (47.5)
*a*	*Ceftriaxone*	14 (24.5)
*b*	*Azithromycin*	06 (10.5)
*c*	*Metronidazole*	05 (08.7)
*d*	*Ciprofloxacin*	03 (05.2)
*e*	Others	*29 (50.8)*
2	Antineoplastic agents	14 (11.6)
*a*	*Cisplatin*	04 (28.5)
*b*	*Cyclophosphamide*	02 (14.2)
*c*	*Others*	08 (57.1)
3	Corticosteroids	11 (09.1)
*a*	*Hydrocortisone*	05 (45.4)
*b*	*Prednisolone*	02 (18.1)
*c*	*Others*	04 (36.3)
4	β2 receptor agonist	09 (07.5)
*a*	*Salbutamol*	09 (07.5)
5	Diuretics	08 (06.6)
*a*	*Furosemide*	06 (75.0)
*b*	*Hydrochlorothiazide*	02 (25.0)
*6*	Others	21 (17.5)

In our study, ceftriaxone (24.5%) and azithromycin (10.5%) were frequently suspected AMAs to induce hypokalemia (Table 2). Out of 27 ICSRs with suspected AMA-induced hypokalemia, nine patients were aged between 21 years and 40 years and majority (74.19%) of these was females. Out of 27 ICSRs, three (11.1%) patients experienced serious adverse events as their hospital stay was prolonged but their severity of hypokalemia was moderate and did not require and instruct to correct it. The causality assessment for all suspected AMAs was possible.

**Table 2:** Analysis of 27 ICSRs containing 53 AMAs for suspected adverse drug reactions

*AMAs (n)*	*Route of administration*		*Severity of hypokalemia*			*Outcome*		*Causality assessment*
	*IV*	*PO*	*Mild*	*Moderate*	*Severe*	*Recovered*	*Continued*	
Ceftriaxone (14)	13	00	10	03	01	02	12	Possible
Azithromycin (06)	01	05	05	01	Nil	01	05	Possible
Metronidazole (05)	03	02	02	03	Nil	00	05	Possible
Ciprofloxacin (03)	02	01	Nil	03	Nil	00	03	Possible
Others (29)	13	16	12	16	01	01	28	Possible

**Table 3:** Two ICSRs with suspected severe hypokalemia due to concomitant medications prescribed with AMA (Total ICSRs-6)

*S. No.*	*AMA*	*Concomitant drug(s)*	*Serum K+ level mEq/L*	*Action taken*	*Indication*	*Outcome*	*Causality assessment*
1	Amphotericin B	Dexamethasone	1.5	Amphotericin B stopped Hypokalemia managed with KCl	Kala azar	Recovered	Possible
2	Ceftriaxone	Prednisolone, Furosemide	2	Ceftriaxone dose reduced Hypokalemia managed with KCl	Nephrotic syndrome	Recovered	Possible

In 14(53.8%), 11(40.7%), and 02(7.4%) ICSRs, the severity of hypokalemia was mild, moderate, and severe, respectively. The severity of hypokalemia was of higher intensity when AMAs were administered parentally than orally. In three ICSRs with moderate to severe hypokalemia were managed by potassium supplementation. In six ICSRs, the possible reason of hypokalemia was drug–drug interaction of AMAs like ceftriaxone, azithromycin, amphotericin B, and vancomycin with concomitant medications like furosemide, hydrocortisone, dexamethasone, and deriphyllin. The severity of hypokalemia in these cases ranged from mild to severe (Table 3).

## DISCUSSION

The potassium loss from the urinary tract, hypomagnesemia, ketonuria, bicarbonaturia, renal tubular acidosis, hyperaldosteronism, and drugs can cause hypokalemia in patients.^11^ Patients with mild hypokalemia are usually asymptomatic,^12^ whereas patients with moderate-to-severe hypokalemia present with generalized weakness, cardiac arrhythmias and acute respiratory failure, hepatic encephalopathy, etc.^13^ In our study, 0.9% of the patients who required AMAs developed hypokalemia. Though the prevalence of hypokalemia in admitted patients irrespective of the cause has been reported to be varying between 3.52^14^ and 20%^15^ which is high.

In this study, six ICSRs had mid to severe grade of hypokalemia as AMAs viz. ceftriaxone and azithromycin were coadministered with hydrocortisone, furosemide, and or deriphyllin. These concomitantly administered drugs are established to cause hypokalemia (Table 3). Due to retrospective nature of this analysis, it was not possible to retrieve the information on the other associated causes of potassium loss like diarrhea, hyperaldosteronism, and serum acid–base balance imbalance.

In a report vancomycin also induced progressive *K*^+^ reductions.^16^ Further, its use concomitantly with furosemide for the treatment of infection at site of amputation,^6^ rifampicin in combination with other antitubercular drugs for treatment of vertebral brucellosis,^17^ sodium penicillin for treatment of coxitis,^18^ flucloxacillin for treatment of spondylodiskitis,^19^ and piperacillin/tazobactam for prophylaxis after hip fracture^20^ was associated with mild to severe intensity of hypokalemia. Recently, National Coordination Centre–Pharmacovigilance Programme of India (NCC–PvPI), Central Drugs Standard Control Organization (CDSCO), Ministry of Health and Family Welfare, Government of India has issued a drug alert that the administration of piperacillin-tazobactam may cause hypokalemia.^21^ Several researchers have reported the association of ceftriaxone, azithromycin, and ciprofloxacin with the development hypokalemia by increasing the urinary potassium excretion.^22–24^

This finding attracts the attention of healthcare professionals to plan focused pharmacovigilance of patients receiving AMAs by monitoring serum K^+^ levels and any sign and symptoms of hypokalemia especially when administered parentally.

## CONCLUSION

Antimicrobial agent induced hypokalemia was frequent in females. Mild hypokalemia was more common in patients taking AMAs only. Severe hypokalemia was prevalent in patients receiving concomitant medications known to cause hypokalemia. This signal needs further investigation.
